# Invisible barriers and lasting scars for racially/ethnically minoritized youth: The role of state‐level opportunity on adverse childhood experiences exposure risk

**DOI:** 10.1002/jts.70085

**Published:** 2026-06-02

**Authors:** Bella C. King, Cynthia M. Navarro Flores, Alejandro L. Vázquez, Jimena Mendoza Barrón, Roberto Obregon Garcia

**Affiliations:** ^1^ Department of Psychology and Neuroscience The University of Tennessee Knoxville Tennessee USA

## Abstract

Previous research has shown there are racial/ethnic disparities in exposure to adverse childhood experiences (ACEs) that can affect youths’ physical and mental well‐being. Structural opportunity factors—including a lack of economic, community, educational, and health resources—may increase exposure to ACEs among youth of color relative to non‐Hispanic White youth. This study explored connections between state‐level opportunity and ACE exposures and examined differences by race/ethnicity within the United States (*N* = 46,395). Average cumulative ACEs were generally significantly higher among Hispanic and non‐Hispanic Black youth relative to non‐Hispanic White youth, *p* < .001. Non‐Hispanic Black youth had higher average cumulative ACEs scores within 28 U.S. states relative to non‐Hispanic White youth. Hispanic youth had higher ACE exposures in 18 states relative to non‐Hispanic White youth. States with higher opportunity scores were in the U.S. Northeast and Midwest. Multilevel model analyses showed that as state‐level opportunity increased (i.e., education, healthcare, economic, community), youth's exposure to ACEs decreased significantly for non‐Hispanic White youth but not non‐Hispanic Black, *p* = .031, or Hispanic youth, *p* = .002, *r* ^2^_conditional_ = .059, who, on average, experienced more ACEs. The findings suggest a structural exclusion of minoritized youth from potential macro‐level protective factors (e.g., high state‐level opportunity), highlighting a need for systemic interventions to decrease racial/ethnic disparities in ACE exposures. The results align with previous research demonstrating that the historical and institutional effects of discrimination may shape disparities in accessing and benefiting from quality education, health care, economic, and community resources.

Adverse childhood experiences (ACEs), such as family instability, community violence, and unmet basic needs, are associated with an increased risk of poor physical and mental health among youth (Liming & Grube, 2018; Swedo et al., 2024). However, the burden of ACE exposure is not equally distributed within the United States. Studies have shown that non‐Hispanic Black and Hispanic youth are more likely than non‐Hispanic White youth to experience ACEs (Mersky et al., 2021). Furthermore, research has highlighted that Hispanic and non‐Hispanic Black youth in the United States are at higher risk of reporting exposure to a larger number of ACEs relative to their non‐Hispanic White counterparts (Maguire‐Jack et al., 2020). Researchers have theorized that these racial/ethnic disparities in adversity exposure may result from distal structural inequities, such as *structural racism*, that contribute to economic hardship, as well as unequal access to healthcare and education among communities of color (Bravemen & Parker Dominguez et al., 2021; Ortiz, 2021). Structural racism refers to the normalization of inequities within institutions and policies that systemically disadvantage racially and ethnically marginalized groups (Bravemen & Parker Dominguez, 2021). However, the current literature largely focuses on more proximal community‐level influences on ACEs exposure disparities among youth, which limits the field's understanding of more distal state‐level influences potentially underpinning these associations (Slopen et al., 2016; Wolff et al., 2018).

Research examining ACEs often uses indicators that represent exposure to childhood abuse or neglect, material scarcity, a caregiver's divorce or separation, the death or incarceration of a caregiver, witnessing domestic or community violence, and living with individuals with a mental health or substance use disorder (Swedo et al., 2024). These experiences are common among youth in the United States, with 76.1% of youth experiencing one or more ACEs during their lifetime (Swedo et al., 2024). The impact of ACE exposures on youth health outcomes can be understood through *life course theory*, which posits that the accumulation of exposures to adversity across a youth's life has an additive effect that increases the risk of adverse health outcomes (Kuh et al., 2003). Research to date has documented this dose–response relationship, with findings indicating that higher numbers of ACE exposures are associated with worse academic, social development, and physical and mental health outcomes among youth (Liming & Grube, 2018; Swedo et al., 2024). As a result, racial/ethnic disparities in adversity exposure may contribute to health disparities documented among marginalized groups (Meléndez Guevara et al., 2024).

Experiencing interpersonal racism has been identified as a risk for ACE exposures, but research has yet to explore broader structural forms of discrimination that contribute to inequities in exposure risk and related health outcomes (Bernard et al., 2022). Racial/ethnic differences in ACE exposures among marginalized youth may be partially explained by the effects of structural racism, which can prevent youth of color from fully obtaining the protective effects of economic, educational, community, and health resources due to inequities perpetuated by institutions and policies in these domains (Slopen et al., 2016; Wolff et al., 2018). These inequities contribute to health‐harming exposures and experiences, including higher levels of exposure to environmental hazards, chronic stressors, and limited access to adequate educational opportunities and health care (Bravemen & Parker Dominguez, 2021). Despite this potential connection between broader structural racism and ACE exposure risk, there is limited research on whether differences in access to state‐level opportunities among minoritized youth contribute to disparities in ACE exposure risk (Ortiz, 2021).

Prior research has documented the associations among economic, educational, community, and health resources; ACE exposures; and related health outcomes. Wolff et al. (2018) examined clusters of ACEs typologies in relation to community‐level risk factors and found that neighborhood poverty, unemployment rate, low rates of educational attainment, and higher dependence on public assistance were associated with higher odds of youth fitting high‐ACE profiles. Their findings also suggest that youths living in more affluent neighborhoods were less likely to be in high‐ACE profile groups. Prior research has also highlighted that structural racism in school policies (e.g., disproportionate suspensions, expulsions, and behavioral referrals) contributes to higher levels of ACE exposures among students of color (Danforth et al., 2024) and that structural racism in pediatric mental health services creates inequities in access to care for non‐Hispanic Black and Hispanic youth (Ortega & Roby, 2021). Although prior studies have demonstrated how state‐level opportunity factors contribute to inequities that can affect youth well‐being (Polanco‐Roman et al., 2025), research to date has not examined potential associations between state‐level opportunity and racial/ethnic differences in the risk of ACE exposures (Ortiz, 2021).

The present study aimed to (a) examine the state‐level distribution of opportunities and ACE disparities between non‐Hispanic White youth and youth of color, (b) assess associations between state‐level opportunity and the risk of cumulative ACE exposures, and (c) examine whether these potential associations were moderated by youth race/ethnicity. Informed by previous research, we hypothesized that higher state‐level opportunity (i.e., educational, health care, economic, and community resources) would be associated with lower levels of ACE exposures overall but that this protective association would be weaker for non‐Hispanic Black and Hispanic youth, who, on average, were expected to report higher exposure to ACEs relative to non‐Hispanic White youth.

## METHOD

### Participants and procedure

Individual‐level data were from 46,395 youth from across the United States who completed the 2022 National Survey of Children's Health (NSCH) between July 2022 and January 2023 (Child and Adolescent Health Measurement Initiative [CAHMI], 2024). The present study used data from youth who were identified as non‐Hispanic White (*n* = 34,733), non‐Hispanic Black (*n* = 3,292), or Hispanic (*n* = 8,370). The NSCH is an annual nationally representative household survey assessing the physical and emotional health of youth aged 0–17 years. Households were randomly selected from the Census Master Address File to ensure balanced participation across states. Eligible households with at least one child aged 0–17 years were invited to participate, and caregivers completed a 36‐min survey online, on paper, or by phone in English or Spanish. As the NSCH is conducted as a needs assessment, formal informed consent was not obtained. Survey materials clearly explained to participants the aims and nature of the survey, stating that participation was completely voluntary and confidential. Analyses of the deidentified 2022 NSCH survey data and opportunity index were approved by the University of Tennessee, Knoxville Institutional Review Board as nonhuman subjects research.

### Measures

#### Demographic information

Caregivers reported their age, sex (male or female), relationship to the child, and level of educational attainment. Caregivers also reported their child's age, sex, and race/ethnicity.

#### ACE exposure

Caregiver reports on seven ACE indicators were used for the present study and included: difficulty meeting basic needs due to family income; parental separation or divorce; the death of a parent or guardian; parental incarceration; witnessing or hearing physical violence between adults in the home; being a victim of violence or witnessing neighborhood violence; and living with someone experiencing mental illness, suicidality, or severe depression; or living with someone with alcohol or drug problems (CAHMI, 2024). ACEs items were coded dichotomously (1 = yes, 0 = no). Several of these ACEs were assessed in the original ACEs study by Felitti et al. (1998; i.e., separation or divorce, incarceration, domestic violence, household member with mental illness, suicidality, depression, and alcohol and drug problems). Our study included additional indicators focusing on basic needs, the death of a caregiver, and violence in the neighborhood, which are also recognized as ACEs within the literature (Karatekin et al., 2022; Folk et al., 2023). Responses were summed to create a cumulative ACEs score, with higher values representing higher levels of exposure to adversity. The ACEs index demonstrated acceptable internal consistency in this sample, Cronbach's α = .70.

#### State‐level opportunity

The Opportunity Index was used to provide scores for all 50 states within the United States. This index is composed of four subscales representing economic, educational, community, and health opportunities (Rackers et al., 2019). The overall index was calculated by averaging the four subscale scores, with higher scores depicting higher state‐level opportunity. Data on state‐level opportunity indices for 2022 were merged with the 2022 NSCH data by the youth's state of residence. Although the Opportunity Index does not provide the individual indicators that compose its overall and composite scales, internal consistency based on the four subscales was acceptable, Cronbach's α = .68.

## RESULTS

The average caregiver age was 24.90 years (*SD* = 9.55), and caregivers predominantly identified as women (*n* = 30,043; 70.2%), were biological caregivers (*n* = 39,306; 91.8%), were married (*n* = 33,232; 77.6%), and had a bachelor's degree (*n* = 13,204; 30.8%). Youth were, on average, 9.64 years old (*SD* = 5.31), and just over half were boys (*n* = 22,129; 51.7%). Within the NSCH sample, 5.5% of responses were missing, and youth who reported higher ACE exposure were more likely to have complete data than those who reported fewer exposures, *p* < > .001–*p* = .039, suggesting data were not missing at random. Missing values were imputed using the random forest algorithm. State‐level opportunity was then visualized for all 50 U.S. states (See Figure 1). States with higher opportunity scores clustered in the Northeast and Midwest regions, whereas lower scores were concentrated in the South. Minnesota had the highest state‐level opportunity score, and Mississippi had the lowest (See Supplementary Table S1). Cumulative ACEs were generally significantly higher among Hispanic (*M* = 0.77, *SD* = 1.24) and non‐Hispanic Black youth (*M* = 0.90, *SD* = 1.25) relative to non‐Hispanic White youth (*M* = 0.61, *SD* = 1.17), *p* < .001. The most frequently reported individual ACEs were living with a parent or guardian who was divorced or separated, difficulties with basic needs (e.g., food, housing), and living with someone with a mental health or substance use problem (i.e., drug or alcohol). Caregivers of non‐Hispanic Black youth were significantly more likely to report the majority of ACE indicators, *p* < .001, except for living with anyone with a mental health problem, *p* = .001, or an alcohol or drug problem, *p* < .001, which were more frequently endorsed among caregivers of non‐Hispanic White youth. See Supplementary Table S2 for the frequencies of individual ACEs and differences by racial/ethnic group.

**FIGURE 1:**
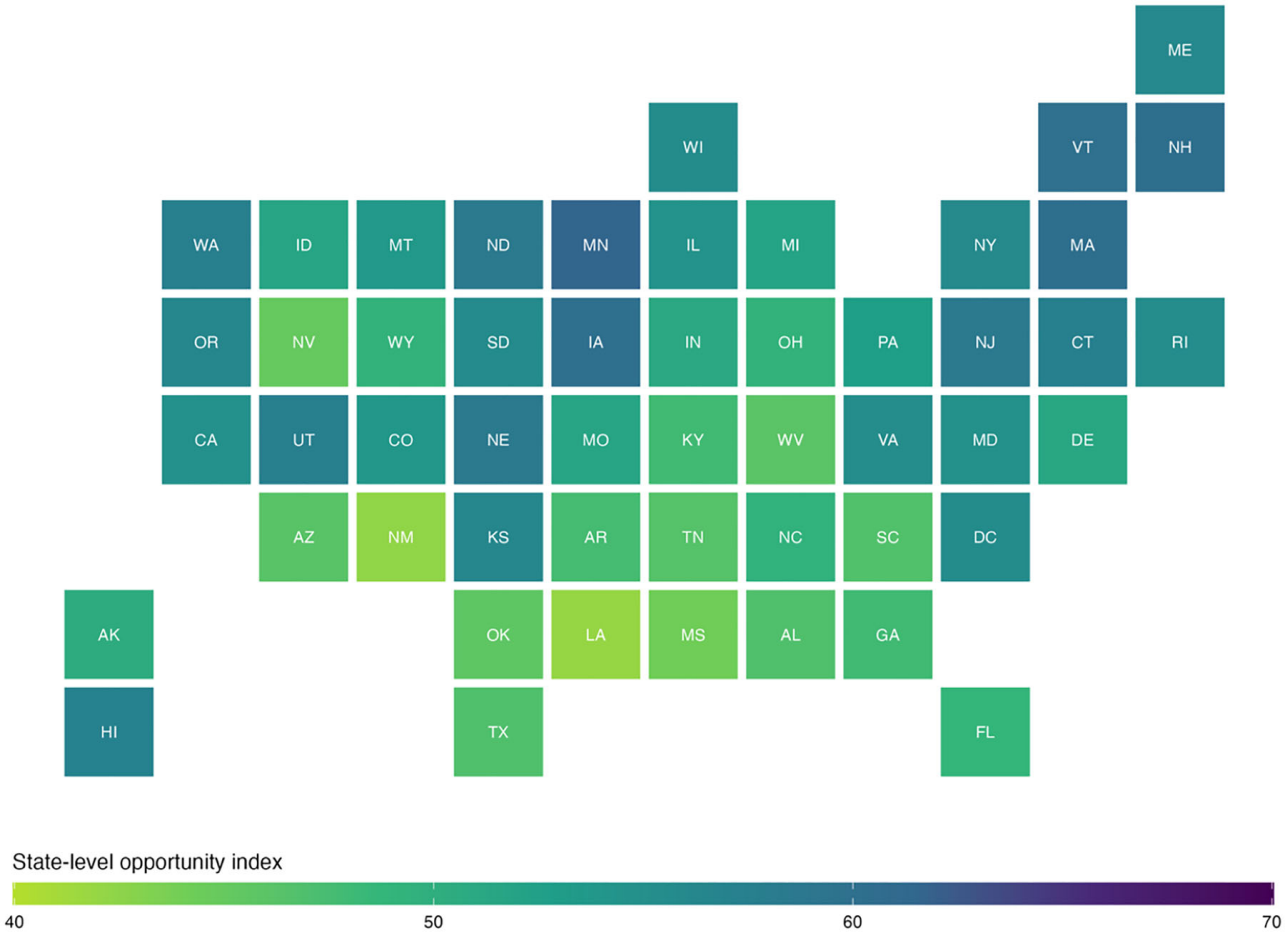
State‐level opportunity index scores across the United States

Relative to non‐Hispanic White youth, non‐Hispanic Black youth had higher cumulative ACEs scores in 28 states, and Hispanic youth had higher scores in 18 states (see Supplementary Table S3). Arkansas was the only state where non‐Hispanic White youth had significantly more adversity exposure relative to Hispanic youth. Bivariate Spearman correlations suggest that state‐level opportunity was negatively associated with cumulative ACEs in the overall sample, ρ = −.68, *p* < .001. However, increases in state‐level opportunity were associated with a moderate decrease in ACEs scores for non‐Hispanic White, ρ = −.57, *p* < .001, and non‐Hispanic Black youth, ρ = −.38, *p* = .007, but not Hispanic youth, ρ = −.08, *p* = .592. An increase in age was associated with higher cumulative ACEs scores, ρ = .24, *p* < .001. Youth sex was not associated with an increased risk of ACE exposure. Quartiles representing the bottom 25%, 25%–50%, 50%–70%, and top 25% state‐level opportunity index scores were calculated. States with opportunity index scores in the bottom 25% quartile had the highest average cumulative ACEs scores in the present sample relative to other quartiles, *p* < .001. Specific individual ACEs were significantly more likely to be reported in states with opportunity index scores within the bottom 25% quartile relative to other quartiles (i.e., difficulties with basic needs like food/housing; parent or guardian divorced or separated, died, or went to jail or prison; or were a victim or witnessed neighborhood violence; living with anyone with alcohol/drug problems), *p* < .001–*p* = .001. (see Supplementary Table S4). Living with anyone with mental health problems was significantly more common among youths living in states with opportunity index scores that were within the top 25%, *p* < .001. Experiencing or witnessing neighborhood violence was not significantly associated with state‐level opportunity index quartiles. The associations between state‐level opportunity index quartiles and individual ACEs followed a similar pattern for non‐Hispanic White youth (Supplementary Table S5). However, only specific ACEs varied by state‐level opportunity index quartiles among non‐Hispanic Black (i.e., parent or guardian served time in jail or prison, experiencing or witnessing neighborhood violence; Supplementary Table S6) and Hispanic youth (i.e., parent or guardian served time in jail or prison, living with anyone with a mental health problem; Supplementary Table S7).

ACEs represented within the cumulative score were largely consistent across groups. ACEs representing difficulties with basic needs (e.g., food, housing) and having a parent or guardian who was divorced or separated were the experiences that were most strongly and consistently correlated with higher ACEs scores across racial/ethnic groups. See Supplementary Table S8 for overall sample and race/ethnicity‐specific Spearman bivariate correlations between cumulative and individual ACE indicators.

Multivariate regression models were then fitted in a stepwise manner, using the maximum likelihood estimator, to determine whether accounting for nesting and random slopes and intercepts improved model fit based on lower Akaike information criterion (AIC) and Bayesian information criterion (BIC) values and statistically significant likelihood ratio tests (Hox et al., 2017). A random‐intercepts multilevel model fit the data significantly better, AIC = 147,862.18, BIC = 147,888.41, than the freely estimated generalized least squares model, AIC = 148,046.43, BIC = 148,063.92, according to relative fit indices and a significant likelihood ratio test, *p* < .001. The random‐intercepts and slopes model demonstrated worse model fit relative to the random‐intercepts model, AIC = 147,866.18, BIC = 147,909.90, but did not significantly differ according to a likelihood ratio test, *p* = 1.00.

The final main effects multilevel model was then fitted using the restricted maximum likelihood estimator, controlling for child age, and indicated that higher state‐level opportunity was significantly associated with lower cumulative ACE exposure among youth overall, *p* < .001 (Table 1), accounting for approximately 5.9% of the conditional variance in ACE exposures (Table 2; Hox et al., 2017). Race/ethnicity was also a significant predictor, with non‐Hispanic Black and Hispanic youth reporting higher cumulative ACEs scores relative to non‐Hispanic White youth, *p* < .001. In the interaction model, the association between state‐level opportunity and ACEs exposure was significantly moderated by race/ethnicity for both Hispanic, *p* = .002, and non‐Hispanic Black youth relative to non‐Hispanic White youth, *p* = .031 (Figure 2). Simple slopes analyses revealed that the protective association between opportunity and ACEs was significant only among non‐Hispanic White youth, *p* < .001 (Table 3).

**TABLE 1:** Multilevel model estimates for associations between youth adverse childhood experiences (ACEs), state‐level opportunity, and race/ethnicity

Variable	Main‐effects model			Interaction model		
	Est.	*SE*	*p*	Est.	*SE*	*p*
Child age	0.048	0.001	< .001	0.048	0.001	< .001
Hispanic^a^	0.160	0.015	< .001	−0.356	0.169	.035
Non‐Hispanic Black^a^	0.263	0.022	< .001	−0.224	0.226	.032
SO	−0.012	0.002	< .001	−0.014	0.003	< .001
Hispanic x SO^a^				0.010	0.003	.002
Non‐Hispanic Black x SO^a^				0.009	0.004	.031

*Note*: *N* = 46,395. SO = state‐level opportunity.

^a^ Relative to non‐Hispanic White.

**TABLE 2:** Model fit statistics

Model	AIC	BIC	Log‐likelihood	Conditional *R* ^2^	Marginal *R* ^2^
Main‐effects	145,437.754	145,498.968	−72,711.877	.059	.044
Interaction	145,447.816	145,526.52	−72,714.908	.059	.044

*Note*: AIC = Akaike information criterion; BIC = Bayesian information criterion.

**FIGURE 2:**
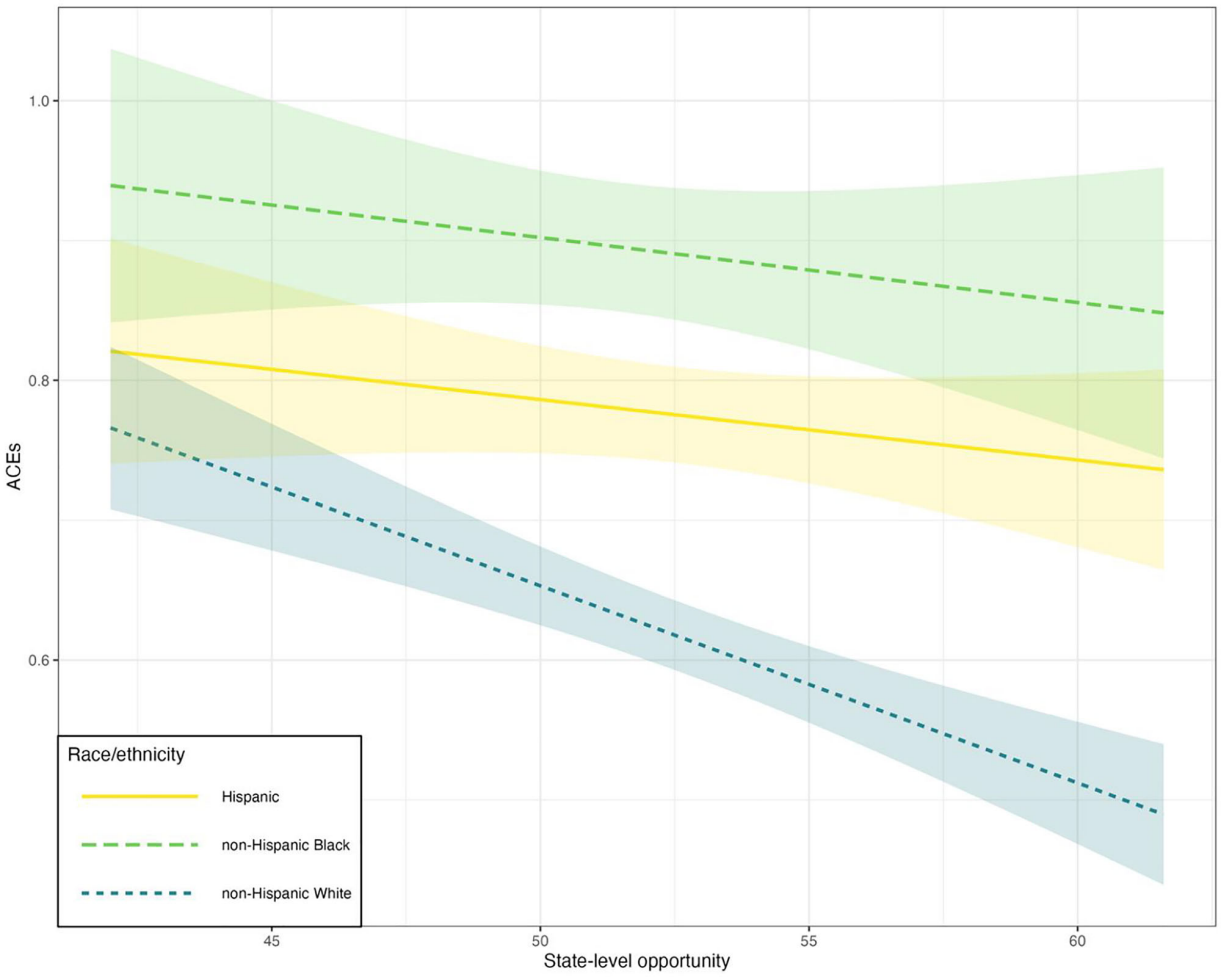
Interaction between state‐level opportunity index scores and youth race/ethnicity in relation to adverse childhood experiences

**TABLE 3:** Between‐group differences

Group	Estimate	*SE*	*t*	*p*
Hispanic	−.004	.004	−1.223	.221
Non‐Hispanic Black	−.005	.005	−0.994	.320
Non‐Hispanic White	−.014	.003	−5.629	< .001

## DISCUSSION

The present study contributes to the literature by extending prior research identifying interpersonal racism as a risk factor for ACEs through the examination of distal structural forms of discrimination that may contribute to increased exposure risk (i.e., inequitable access to resources and opportunities; Bernard et al., 2022). Consistent with prior research, non‐Hispanic Black and Hispanic youth had higher average cumulative ACEs scores relative to non‐Hispanic White youth (Maguire‐Jack et al., 2020; Slopen et al., 2016). Our findings also suggest that cumulative ACEs were significantly higher across multiple states for Hispanic and non‐Hispanic Black youth relative to non‐Hispanic White youth, suggesting that the burden of ACEs is unequally distributed geographically between racial/ethnic groups (Mersky et al., 2021). The use of a state‐level opportunity index identified specific geographic regions with higher economic, educational, health, and community resources. States with higher state‐level opportunity scores were primarily located in the Northeast and Upper Midwest regions of the United States. Although these areas may have stronger economies due to a history of industrialization and large population centers, the protective effects of structural opportunity might be restricted for marginalized youth in these contexts due to more structural racial/ethnic segregation relative to other contexts commonly associated with racism (e.g., southern U.S. states; Siegel et al., 2023).

Bivariate correlations suggested that higher state‐level opportunity was associated with generally lower levels of ACE exposures. However, multivariate analyses elucidated that higher state‐level opportunity was only significantly associated with lower ACEs scores for non‐Hispanic White youth and not for non‐Hispanic Black and Hispanic youth. Individual ACEs were frequently associated with lower state‐level opportunity index scores, and representation within the cumulative ACE scores was largely consistent across racial/ethnic groups. Collectively, these findings suggest that although greater state‐level opportunity is generally linked to lower adversity exposure, racial/ethnic disparities persist even in states with higher levels of opportunity, which extends research on community‐level correlates of ACE exposure risk (Wolff et al., 2018).

Although the effect sizes for the multivariate analyses in this study are small, the findings have important theoretical implications for understanding how structural factors may shape ACE exposure risk among youth (Ortiz, 2021). The findings may be explained by structural exclusion from access to and benefit from living in high‐resource states for racially/ethnically marginalized youth (Camacho & Henderson, 2022; Navarro Flores et al., 2026). This is consistent with critical theories positing that structural racism creates inequitable access to resources and opportunities that disproportionately benefit the dominant group (Bravemen & Parker Dominguez, 2021). These disparities in ACEs exposure risk may convey health advantages for non‐Hispanic White youth by reducing their risk of developing related physical and mental health disorders relative to marginalized youth (Bravemen & Parker Dominguez, 2021; Liming & Grube, 2018). Our study did not demonstrate an association between the opportunity index and a lower risk of ACE exposures among marginalized youth, but this should not be interpreted as suggesting that contextual resources do not protect against ACE exposure risk within this population. Prior research has documented that marginalized youth living in affluent communities with access to resources and opportunities are at a reduced risk for ACE exposures relative to those living in disadvantaged communities (Wolff et al., 2018). Our findings build upon this work by documenting that potential distal protective effects of living in high‐resource states may not trickle down to minoritized communities, emphasizing the importance of targeted community‐based interventions to reduce adversity exposure risk (Navarro Flores et al., 2026; Camacho & Henderson, 2022). Thus, these findings suggest that the presence of structural opportunity at the state level may not fully buffer the effects of adversity for marginalized youth, as barriers to accessing or benefiting from these resources may limit their protective impact.

Our findings highlight a need for research aiming to identify and address policies that contribute to racial/ethnic disparities in accessing and benefiting from the potential protective effects of state‐level opportunities (Ortiz, 2021). The results of this study indicate that although distal factors like state‐level opportunity have a small association with ACE exposure, risk may be more indicative of proximal community factors (e.g., community disadvantage, resource availability; Wolff et al., 2018). To advance this work, there is a need to increase state‐level participation and availability of health behavior surveillance systems that allow for analyses of county or census tract–level data that are closer in proximity to the youth (Swedo et al., 2024). The findings from this study are limited by the nature of the data, which are cross‐sectional; thus, these findings should be considered structural correlates of ACE exposure risk. Future research should explore a broader array of ACEs beyond the limited number available in the NSCH dataset (Karatekin et al., 2022; Folk et al., 2023). Longitudinal research is also needed to confirm and clarify associations among state‐level opportunity, race/ethnicity, and ACE exposures across time.

## OPEN PRACTICES STATEMENT

The present study was not formally pre‐registered. Data can be retrieved through the following link for the National Survey of Children's Health: https://www.census.gov/programs‐surveys/nsch.html. Survey questionnaires and data manuals are also publicly available. Data for the Opportunity Index can be retrieved through the following link: https://opportunityindex.org/. The R code used for the present study will be provided upon request to the corresponding author.

## AUTHOR CONTRIBUTIONS

**Bella C. King**: writing—original draft, conceptualization, writing—review and editing. **Cynthia M. Navarro Flores**: conceptualization, methodology, formal analysis, writing—original draft, supervision, writing—review and editing, visualization. **Alejandro L. Vázquez**: conceptualization, methodology, formal analysis, supervision, visualization, writing—original draft, writing—review and editing. **Jimena Mendoza Barrón**: writing—original draft, writing—review and editing. **Roberto Obregon Garcia**: writing—original draft, writing—review and editing.

## Supporting information



Supporting Information
