# Supplementary material for: Invisible barriers and lasting scars for racially/ethnically minoritized youth: The role of state‐level opportunity on adverse childhood experiences exposure risk
Source: J Trauma Stress. 2026 Jun 2;39(5):975–82. doi: 10.1002/jts.70085 (PMC13639205; doi:10.1002/jts.70085)
Supplement: Supplementary file 1 [file JTS-39-975-s001.docx]

| **Supplemental Table S1**  *State-level Opportunity and Youth Race/Ethnicity* | | | | | |
| --- | --- | --- | --- | --- | --- |
| State | Opportunity | Sample | | | |
|  | index | Overall | Hispanic | non-Hispanic Black | non-Hispanic White |
|  |  | (*n* = 46,395) | (*n* = 8,370) | (*n* = 3,292) | (*n* = 34,733) |
| Alabama | 46.8 | 702 (1.5%) | 31 (0.4%) | 126 (3.8%) | 545 (1.6%) |
| Alaska | 50.4 | 510 (1.1%) | 65 (0.8%) | 16 (0.5%) | 429 (1.2%) |
| Arizona | 46.2 | 702 (1.5%) | 265 (3.2%) | 21 (0.6%) | 416 (1.2%) |
| Arkansas | 47.6 | 745 (1.6%) | 81 (1%) | 66 (2%) | 598 (1.7%) |
| California | 54.9 | 3788 (8.2%) | 1759 (21%) | 79 (2.4%) | 1950 (5.6%) |
| Colorado | 53.9 | 1470 (3.2%) | 324 (3.9%) | 30 (0.9%) | 1116 (3.2%) |
| Connecticut | 57.4 | 721 (1.6%) | 140 (1.7%) | 53 (1.6%) | 528 (1.5%) |
| Delaware | 51.3 | 626 (1.3%) | 81 (1%) | 111 (3.4%) | 434 (1.2%) |
| Florida | 48.9 | 653 (1.4%) | 248 (3%) | 51 (1.5%) | 354 (1%) |
| Georgia | 47.9 | 1068 (2.3%) | 160 (1.9%) | 262 (8%) | 646 (1.9%) |
| Hawaii | 57.2 | 236 (0.5%) | 103 (1.2%) | 4 (0.1%) | 129 (0.4%) |
| Idaho | 51.7 | 796 (1.7%) | 109 (1.3%) | 4 (0.1%) | 683 (2%) |
| Illinois | 54.6 | 672 (1.4%) | 125 (1.5%) | 47 (1.4%) | 500 (1.4%) |
| Indiana | 50.9 | 729 (1.6%) | 76 (0.9%) | 50 (1.5%) | 603 (1.7%) |
| Iowa | 60.1 | 792 (1.7%) | 47 (0.6%) | 30 (0.9%) | 715 (2.1%) |
| Kansas | 56.4 | 727 (1.6%) | 116 (1.4%) | 14 (0.4%) | 597 (1.7%) |
| Kentucky | 47.9 | 746 (1.6%) | 51 (0.6%) | 35 (1.1%) | 660 (1.9%) |
| Louisiana | 42.0 | 729 (1.6%) | 75 (0.9%) | 151 (4.6%) | 503 (1.4%) |
| Maine | 56.0 | 797 (1.7%) | 24 (0.3%) | 9 (0.3%) | 764 (2.2%) |
| Maryland | 54.8 | 623 (1.3%) | 89 (1.1%) | 132 (4%) | 402 (1.2%) |
| Massachusetts | 60.3 | 632 (1.4%) | 80 (1%) | 35 (1.1%) | 517 (1.5%) |
| Michigan | 51.9 | 733 (1.6%) | 64 (0.8%) | 52 (1.6%) | 617 (1.8%) |
| Minnesota | 61.6 | 728 (1.6%) | 45 (0.5%) | 41 (1.2%) | 642 (1.8%) |
| Mississippi | 44.2 | 643 (1.4%) | 37 (0.4%) | 186 (5.7%) | 420 (1.2%) |
| Missouri | 51.6 | 753 (1.6%) | 50 (0.6%) | 57 (1.7%) | 646 (1.9%) |
| Montana | 53.6 | 689 (1.5%) | 35 (0.4%) | 0 (0%) | 654 (1.9%) |
| Nebraska | 58.5 | 900 (1.9%) | 113 (1.4%) | 29 (0.9%) | 758 (2.2%) |
| Nevada | 45.0 | 557 (1.2%) | 215 (2.6%) | 29 (0.9%) | 313 (0.9%) |
| New Hampshire | 60.2 | 705 (1.5%) | 62 (0.7%) | 8 (0.2%) | 635 (1.8%) |
| New Jersey | 58.5 | 586 (1.3%) | 157 (1.9%) | 50 (1.5%) | 379 (1.1%) |
| New Mexico | 42.3 | 751 (1.6%) | 451 (5.4%) | 14 (0.4%) | 286 (0.8%) |
| New York | 55.9 | 3793 (8.2%) | 898 (10.7%) | 395 (12%) | 2500 (7.2%) |
|  | | | | | |

| **Supplemental Table S1**  *State-level Opportunity and Youth Race/Ethnicity (Continued)* | | | | | |
| --- | --- | --- | --- | --- | --- |
| State | Opportunity | Sample | | | |
|  | index | Overall | Hispanic | non-Hispanic Black | non-Hispanic White |
|  |  | (*n* = 46,395) | (*n* = 8,370) | (*n* = 3,292) | (*n* = 34,733) |
| North Carolina | 49.5 | 692 (1.5%) | 94 (1.1%) | 88 (2.7%) | 510 (1.5%) |
| North Dakota | 58.5 | 690 (1.5%) | 41 (0.5%) | 19 (0.6%) | 630 (1.8%) |
| Ohio | 49.2 | 1285 (2.8%) | 101 (1.2%) | 120 (3.6%) | 1064 (3.1%) |
| Oklahoma | 45.6 | 558 (1.2%) | 104 (1.2%) | 26 (0.8%) | 428 (1.2%) |
| Oregon | 56.4 | 2017 (4.3%) | 385 (4.6%) | 35 (1.1%) | 1597 (4.6%) |
| Pennsylvania | 52.7 | 1362 (2.9%) | 122 (1.5%) | 64 (1.9%) | 1176 (3.4%) |
| Rhode Island | 55.5 | 675 (1.5%) | 115 (1.4%) | 31 (0.9%) | 529 (1.5%) |
| South Carolina | 46.7 | 696 (1.5%) | 65 (0.8%) | 116 (3.5%) | 515 (1.5%) |
| South Dakota | 55.7 | 759 (1.6%) | 46 (0.5%) | 18 (0.5%) | 695 (2%) |
| Tennessee | 46.4 | 1655 (3.6%) | 140 (1.7%) | 189 (5.7%) | 1326 (3.8%) |
| Texas | 46.8 | 684 (1.5%) | 280 (3.3%) | 73 (2.2%) | 331 (1%) |
| Utah | 57.7 | 733 (1.6%) | 103 (1.2%) | 8 (0.2%) | 622 (1.8%) |
| Vermont | 59.9 | 716 (1.5%) | 19 (0.2%) | 10 (0.3%) | 687 (2%) |
| Virginia | 55.3 | 660 (1.4%) | 101 (1.2%) | 88 (2.7%) | 471 (1.4%) |
| Washington | 57.6 | 587 (1.3%) | 107 (1.3%) | 26 (0.8%) | 454 (1.3%) |
| West Virginia | 46.1 | 786 (1.7%) | 29 (0.3%) | 18 (0.5%) | 739 (2.1%) |
| Wisconsin | 55.6 | 753 (1.6%) | 67 (0.8%) | 27 (0.8%) | 659 (1.9%) |
| Wyoming | 49.2 | 1151 (2.5%) | 161 (1.9%) | 9 (0.3%) | 981 (2.8%) |
|  | | | | | |

| **Supplemental Table S2**  *Frequencies of Individual ACEs and Differences by Racial/Ethnic Group.* | | | | | |
| --- | --- | --- | --- | --- | --- |
| ACEs indicators | Overall | Hispanic | non-Hispanic Black | non-Hispanic White | *p* |
|  | (*N* = 46,395) | (*n* = 8,370) | (*n* = 3,292) | (*n* = 34,733) |  |
| Difficulties with basic needs like food/housing |  |  |  |  | <.001 |
| Yes | 5,394 (11.6%) | 1,370 (16.4%) | 629 (19.1%) | 3,395 (9.8%) |  |
| No | 41,001 (88.4%) | 7,000 (83.6%) | 2,663 (80.9%) | 31,338 (90.2%) | |
| Parent or guardian divorced or separated |  |  |  |  | <.001 |
| Yes | 9,557 (20.6%) | 2,072 (24.8%) | 936 (28.4%) | 6549 (18.9%) | |
| No | 36838 (79.4%) | 6,298 (75.2%) | 2,356 (71.6%) | 28,184 (81.1%) | |
| Parent or guardian died |  |  |  |  | <.001 |
| Yes | 1,299 (2.8%) | 250 (3%) | 208 (6.3%) | 841 (2.4%) |  |
| No | 45,096 (97.2%) | 8,120 (97%) | 3,084 (93.7%) | 33,892 (97.6%) | |
| Parent or guardian served time in jail or prison |  |  |  |  | <.001 |
| Yes | 2,433 (5.2%) | 505 (6%) | 315 (9.6%) | 1,613 (4.6%) |  |
| No | 43,962 (94.8%) | 7,865 (94%) | 2,977 (90.4%) | 33,120 (95.4%) | |
| Witnessed domestic violence |  |  |  |  | <.001 |
| Yes | 2,082 (4.5%) | 448 (5.4%) | 213 (6.5%) | 1,421 (4.1%) |  |
| No | 44,313 (95.5%) | 7,922 (94.6%) | 3,079 (93.5%) | 33,312 (95.9%) | |
| Victim or witness of neighborhood violence |  |  |  |  | <.001 |
| Yes | 1,453 (3.1%) | 354 (4.2%) | 173 (5.3%) | 926 (2.7%) |  |
| No | 44,942 (96.9%) | 8,016 (95.8%) | 3,119 (94.7%) | 33,807 (97.3%) | |
| Lived with anyone with mental health problems |  |  |  |  | <.001 |
| Yes | 4,317 (9.3%) | 711 (8.5%) | 240 (7.3%) | 3,366 (9.7%) |  |
| No | 42,078 (90.7%) | 7,659 (91.5%) | 3,052 (92.7%) | 31,367 (90.3%) | |
| Lived with anyone with alcohol/drug problems |  |  |  |  | .001 |
| Yes | 4,133 (8.9%) | 735 (8.8%) | 238 (7.2%) | 3,160 (9.1%) |  |
| No | 42,262 (91.1%) | 7,635 (91.2%) | 3,054 (92.8%) | 31,573 (90.9%) |  |
| Cumulative ACEs | 0.66 (1.19) | 0.77 (1.24) | 0.90 (1.25) | 0.61 (1.17) | <.001 |
| *Note: p* = *p*-value for Chi-Square Test of Independence. | | | | | |

| **Supplemental Table S3**  *State-level Differences in Average Cumulative ACEs exposure Between Racial/Ethnic Groups.* | | | | | | | | | |
| --- | --- | --- | --- | --- | --- | --- | --- | --- | --- |
| State |  | Cumulative ACEs exposure | | | | | | |  |
|  | Overall | | non-Hispanic White | non-Hispanic Black | Dif | *p* | Hispanic | Dif | *p* |
| Alabama | 0.76 (1.27) | | 0.71(1.29) | 0.92 (1.18) | 0.21 | <.001 | 0.87 (1.34) | 0.16 | .273 |
| Alaska | 0.60 (1.14) | | 0.58 (1.14) | 0.75 (1.13) | 0.17 | .260 | 0.75 (1.16) | 0.18 | .089 |
| Arizona | 0.69 (1.21) | | 0.60 (1.11) | 1.29 (1.14) | 0.69 | .010 | 0.78 (1.30) | 0.18 | .069 |
| Arkansas | 0.90 (1.46) | | 0.96 (1.53) | 0.83 (1.08) | -0.13 | .554 | 0.53 (1.10) | -0.43 | .019 |
| California | 0.74 (1.23) | | 0.73 (1.29) | 1.00 (1.28) | 0.27 | .002 | 0.74 (1.17) | 0.01 | .008 |
| Colorado | 0.69 (1.24) | | 0.61 (1.15) | 0.97 (1.07) | 0.36 | .003 | 0.98 (1.48) | 0.37 | <.001 |
| Connecticut | 0.54 (1.02) | | 0.41(0.92) | 0.92 (1.27) | 0.52 | <.001 | 0.89 (1.16) | 0.49 | <.001 |
| Delaware | 0.69 (1.21) | | 0.64 (1.17) | 0.88 (1.26) | 0.24 | .019 | 0.69 (1.32) | 0.05 | .832 |
| Florida | 0.65 (1.14) | | 0.63 (1.11) | 0.76 (1.03) | 0.13 | .093 | 0.65 (1.20) | 0.01 | .911 |
| Georgia | 0.63 (1.11) | | 0.59 (1.09) | 0.79 (1.20) | 0.20 | <.001 | 0.56 (1.03) | -0.03 | .862 |
| Hawaii | 0.60 (1.21) | | 0.33 (0.73) | 0.25 (0.50) | -0.08 | .949 | 0.95 (1.57) | 0.62 | <.001 |
| Idaho | 0.66 (1.19) | | 0.65 (1.20) | 0.00 (0.00) | -0.65 | .165 | 0.72 (1.15) | 0.07 | .147 |
| Illinois | 0.52 (1.06) | | 0.48 (1.05) | 1.00 (1.35) | 0.52 | <.001 | 0.50 (0.90) | 0.02 | .274 |
| Indiana | 0.70 (1.27) | | 0.66 (1.28) | 0.88 (1.26) | 0.22 | .044 | 0.92 (1.17) | 0.27 | .003 |
| Iowa | 0.63 (1.12) | | 0.60 (1.06) | 0.77 (1.28) | 0.17 | .497 | 1.00 (1.68) | 0.40 | .232 |
| Kansas | 0.63 (1.19) | | 0.57 (1.18) | 0.64 (1.01) | 0.07 | .537 | 0.91 (1.23) | 0.34 | <.001 |
| Kentucky | 0.79 (1.33) | | 0.80 (1.37) | 0.74 (0.98) | -0.05 | .443 | 0.71(1.37) | -0.09 | .578 |
| Louisiana | 0.76 (1.25) | | 0.68 (1.19) | 0.96 (1.46) | 0.28 | .007 | 0.84 (1.21) | 0.16 | .090 |
| Maine | 0.71(1.28) | | 0.71 (1.29) | 0.56 (1.13) | -0.16 | .539 | 0.58 (0.97) | -0.13 | .792 |
| Maryland | 0.52 (0.95) | | 0.46 (0.95) | 0.70 (0.97) | 0.24 | <.001 | 0.55 (0.88) | 0.09 | .124 |
| Massachusetts | 0.56 (1.04) | | 0.52 (1.04) | 0.86 (1.14) | 0.34 | .010 | 0.65 (0.96) | 0.13 | .057 |
| Michigan | 0.66 (1.20) | | 0.60 (1.14) | 1.15 (1.60) | 0.55 | <.001 | 0.78 (1.34) | 0.18 | .221 |
| Minnesota | 0.43 (0.92) | | 0.41(0.90) | 0.63 (0.92) | 0.22 | .009 | 0.58 (1.20) | 0.17 | .302 |
| Mississippi | 0.81 (1.34) | | 0.80 (1.35) | 0.83 (1.20) | 0.04 | .116 | 0.84 (1.74) | 0.04 | .693 |
| Missouri | 0.69 (1.19) | | 0.66 (1.19) | 1.11(1.40) | 0.44 | <.001 | 0.60 (0.90) | -0.06 | .771 |
| Montana | 0.71 (1.31) | | 0.69 (1.27) | - | - | - | 1.09 (1.95) | 0.40 | .280 |
| Nebraska | 0.58 (1.14) | | 0.53 (1.11) | 0.79 (1.37) | 0.26 | .325 | 0.84 (1.29) | 0.31 | .001 |
| Nevada | 0.70 (1.20) | | 0.65 (1.21) | 0.76 (1.09) | 0.11 | .376 | 0.77 (1.20) | 0.12 | .060 |
| New Hampshire | 0.56 (1.03) | | 0.53 (1.00) | 0.38 (0.52) | -0.15 | .882 | 0.87 (1.23) | 0.34 | .007 |
| New Jersey | 0.47 (1.00) | | 0.35 (0.89) | 0.60 (0.81) | 0.25 | <.001 | 0.73 (1.23) | 0.38 | <.001 |
| New Mexico | 0.86 (1.36) | | 0.78 (1.36) | 0.93 (1.44) | 0.15 | .674 | 0.91 (1.36) | 0.13 | .070 |
| New York | 0.61 (1.08) | | 0.52 (1.07) | 0.89 (1.13) | 0.37 | <.001 | 0.73 (1.07) | 0.21 | <.001 |
| *Note:* ACEs = Adverse Childhood Experiences, Dif = Hispanic or non-Hispanic Black ACEs minus non-Hispanic White ACEs, *p* = *p*-value for Kruskal-Wallis Rank Sum Test. | | | | | | | | | |
|  | | | | | |  |  |  |  |

| **Supplemental Table S3**  *State-level Differences in Average Cumulative ACEs exposure Between Racial/Ethnic Groups (Continued).* | | | | | | | | | |
| --- | --- | --- | --- | --- | --- | --- | --- | --- | --- |
| State |  | Cumulative ACEs exposure | | | | | | |  |
|  | Overall | | non-Hispanic White | non-Hispanic Black | Dif | *p* | Hispanic | Dif | *p* |
| North Carolina | 0.60 (1.17) | | 0.52 (1.11) | 0.94 (1.44) | 0.43 | <.001 | 0.72 (1.12) | 0.21 | .001 |
| North Dakota | 0.58 (1.22) | | 0.58 (1.24) | 0.74 (1.28) | 0.16 | .377 | 0.51(0.93) | -0.06 | .701 |
| Ohio | 0.67 (1.26) | | 0.62 (1.23) | 0.99 (1.26) | 0.37 | <.001 | 0.81 (1.53) | 0.19 | .223 |
| Oklahoma | 0.85 (1.41) | | 0.78 (1.36) | 0.96 (1.37) | 0.18 | .188 | 1.09 (1.60) | 0.31 | .009 |
| Oregon | 0.76 (1.30) | | 0.71(1.27) | 1.06 (1.37) | 0.34 | .036 | 0.91(1.42) | 0.20 | .005 |
| Pennsylvania | 0.61(1.12) | | 0.56 (1.08) | 1.23 (1.39) | 0.67 | <.001 | 0.75 (1.18) | 0.19 | .016 |
| Rhode Island | 0.55 (1.02) | | 0.49 (0.99) | 0.61 (0.88) | 0.12 | .245 | 0.77 (1.15) | 0.28 | .002 |
| South Carolina | 0.61 (1.17) | | 0.54 (1.11) | 0.91 (1.31) | 0.37 | <.001 | 0.60 (1.21) | 0.06 | .498 |
| South Dakota | 0.54 (1.08) | | 0.52 (1.05) | 0.89 (1.28) | 0.37 | .025 | 0.78 (1.47) | 0.27 | .181 |
| Tennessee | 0.77 (1.27) | | 0.74 (1.27) | 1.00 (1.38) | 0.26 | .002 | 0.70 (1.10) | -0.04 | .880 |
| Texas | 0.67 (1.24) | | 0.48 (1.06) | 0.99 (1.36) | 0.50 | <.001 | 0.81 (1.37) | 0.33 | <.001 |
| Utah | 0.55 (1.05) | | 0.51 (1.02) | 0.50 (0.76) | -0.01 | .674 | 0.82 (1.17) | 0.31 | .001 |
| Vermont | 0.67 (1.22) | | 0.67 (1.22) | 1.00 (1.33) | 0.33 | .250 | 0.84 (1.30) | 0.18 | .424 |
| Virginia | 0.51(1.03) | | 0.43 (0.95) | 0.82 (1.16) | 0.39 | <.001 | 0.65 (1.20) | 0.23 | .053 |
| Washington | 0.54 (1.05) | | 0.52 (1.02) | 0.58 (1.21) | 0.06 | .550 | 0.64 (1.14) | 0.13 | .340 |
| West Virginia | 0.83 (1.38) | | 0.79 (1.33) | 1.89 (1.94) | 1.10 | .003 | 1.10 (1.92) | 0.31 | .538 |
| Wisconsin | 0.58 (1.14) | | 0.54 (1.12) | 1.07 (1.36) | 0.53 | .006 | 0.72 (1.15) | 0.17 | .129 |
| Wyoming | 0.77 (1.31) | | 0.74 (1.30) | 1.00 (1.22) | 0.26 | .156 | 0.91 (1.36) | 0.17 | .032 |
| *Note:* ACEs = Adverse Childhood Experiences, Dif = Hispanic or non-Hispanic Black ACEs minus non-Hispanic White ACEs, *p* = *p*-value for Kruskal-Wallis Rank Sum Test. | | | | | | | | | |

| **Supplemental Table S4**  *Associations Between Individual ACEs Exposures and State-level Opportunity Quantiles: Overall Sample (N = 46,395).* | | | | | |
| --- | --- | --- | --- | --- | --- |
| ACEs indicators | Bottom 25% | 25% to 50% | 50% to 75% | Top 25% | *p* |
|  | (*n* = 11,675) | (*n* = 12,091) | (*n* = 11,062) | (*n* = 11,567) |  |
| Difficulties with basic needs like food/housing | | | | | <.001 |
| Yes | 1,564 (13.4%) | 1,346 (11.1%) | 1,310 (11.8%) | 1,174 (10.1%) |  |
| No | 10,111 (86.6%) | 10,745 (88.9%) | 9,752 (88.2%) | 10,393 (89.9%) | |
| Parent or guardian divorced or separated |  |  |  |  | <.001 |
| Yes | 2,769 (23.7%) | 2,412 (19.9%) | 2,224 (20.1%) | 2,152 (18.6%) |  |
| No | 8,906 (76.3%) | 9,679 (80.1%) | 8838 (79.9%) | 9,415 (81.4%) |  |
| Parent or guardian died |  |  |  |  | <.001 |
| Yes | 429 (3.7%) | 350 (2.9%) | 290 (2.6%) | 230 (2.0%) |  |
| No | 11,246 (96.3%) | 11,741 (97.1%) | 10,772 (97.4%) | 11,337 (98%) |  |
| Parent or guardian served time in jail or prison |  |  |  |  | <.001 |
| Yes | 781 (6.7%) | 674 (5.6%) | 490 (4.4%) | 488 (4.2%) |  |
| No | 10,894 (93.3%) | 11,417 (94.4%) | 10,572 (95.6%) | 11,079 (95.8%) | |
| Witnessed domestic violence |  |  |  |  | <.001 |
| Yes | 657 (5.6%) | 526 (4.4%) | 427 (3.9%) | 472 (4.1%) |  |
| No | 11,018 (94.4%) | 11,565 (95.6%) | 10,635 (96.1%) | 11,095 (95.9%) | |
| Victim or witness of neighborhood violence |  |  |  |  | .097 |
| Yes | 365 (3.1%) | 366 (3%) | 384 (3.5%) | 338 (2.9%) |  |
| No | 11,310 (96.9%) | 11,725 (97%) | 10,678 (96.5%) | 11,229 (97.1%) | |
| Lived with anyone with mental health problems |  |  |  |  | <.001 |
| Yes | 1,076 (9.2%) | 1,162 (9.6%) | 897 (8.1%) | 1,182 (10.2%) |  |
| No | 10,599 (90.8%) | 10,929 (90.4%) | 10,165 (91.9%) | 10385 (89.8%) | |
| Lived with anyone with alcohol/drug problems |  |  |  |  | .001 |
| Yes | 1,114 (9.5%) | 1,114 (9.2%) | 901 (8.1%) | 1,004 (8.7%) |  |
| No | 10,561 (90.5%) | 10,977 (90.8%) | 10,161 (91.9%) | 10,563 (91.3%) | |
| Cumulative ACEs | 0.75 (1.28) | 0.66 (1.20) | 0.63 (1.13) | 0.61 (1.15) | <.001 |
| *Note:* *p* = *p*-value for Chi-Square Test of Independence. Higher quartiles associated with greater state-level opportunity. | | | | | |

| **Supplemental Table S5**  *Associations Between Individual ACEs Exposures and State-level Opportunity Quantiles: non-Hispanic White (n = 34,733).* | | | | | |
| --- | --- | --- | --- | --- | --- |
| ACEs indicators | Bottom 25% | 25% to 50% | 50% to 75% | Top 25% | *p* |
|  | (*n* = 8,080) | (*n* = 9,815) | (*n* = 7,184) | (*n* = 9,654) |  |
| Difficulties with basic needs like food/housing |  |  |  |  | <.001 |
| Yes | 952 (11.8%) | 969 (9.9%) | 630 (8.8%) | 844 (8.7%) |  |
| No | 7,128 (88.2%) | 8,846 (90.1%) | 6,554 (91.2%) | 8,810 (91.3%) | |
| Parent or guardian divorced or separated |  |  |  |  | <.001 |
| Yes | 1,827 (22.6%) | 1,817 (18.5%) | 1,236 (17.2%) | 1,669 (17.3%) | |
| No | 6,253 (77.4%) | 7,998 (81.5%) | 5,948 (82.8%) | 7,985 (82.7%) | |
| Parent or guardian died |  |  |  |  | <.001 |
| Yes | 264 (3.3%) | 247 (2.5%) | 157 (2.2%) | 173 (1.8%) |  |
| No | 7,816 (96.7%) | 9,568 (97.5%) | 7,027 (97.8%) | 9,481 (98.2%) | |
| Parent or guardian served time in jail or prison |  |  |  |  | <.001 |
| Yes | 500 (6.2%) | 488 (5%) | 261 (3.6%) | 364 (3.8%) |  |
| No | 7,580 (93.8%) | 9,327 (95%) | 6,923 (96.4%) | 9,290 (96.2%) | |
| Witnessed domestic violence |  |  |  |  | <.001 |
| Yes | 435 (5.4%) | 385 (3.9%) | 245 (3.4%) | 356 (3.7%) |  |
| No | 7,645 (94.6%) | 9,430 (96.1%) | 6,939 (96.6%) | 9,298 (96.3%) | |
| Victim or witness of neighborhood violence |  |  |  |  | .654 |
| Yes | 223 (2.8%) | 246 (2.5%) | 200 (2.8%) | 257 (2.7%) |  |
| No | 7,857 (97.2%) | 9,569 (97.5%) | 6,984 (97.2%) | 9,397 (97.3%) | |
| Lived with anyone with mental health problems |  |  |  |  | .018 |
| Yes | 776 (9.6%) | 962 (9.8%) | 635 (8.8%) | 993 (10.3%) |  |
| No | 7,304 (90.4%) | 8,853 (90.2%) | 6,549 (91.2%) | 8,661 (89.7%) | |
| Lived with anyone with alcohol/drug problems |  |  |  |  | .003 |
| Yes | 805 (10%) | 913 (9.3%) | 610 (8.5%) | 832 (8.6%) |  |
| No | 7,275 (90%) | 8,902 (90.7%) | 6,574 (91.5%) | 8,822 (91.4%) | |
| Cumulative ACEs | 0.72 (1.27) | 0.61 (1.18) | 0.55 (1.11) | 0.57 (1.12) | <.001 |
| *Note:* *p* = *p*-value for Chi-Square Test of Independence. Higher quartiles associated with greater state-level opportunity. | | | | | |

| **Supplemental Table S6**  *Associations Between Individual ACEs Exposures and State-level Opportunity Quantiles: non-Hispanic Black (n = 3,292).* | | | | | |
| --- | --- | --- | --- | --- | --- |
| ACEs indicators | Bottom 25% | 25% to 50% | 50% to 75% | Top 25% | *p* |
|  | (*n* = 1,363) | (*n* = 780) | (*n* = 778) | (*n* = 371) |  |
| Difficulties with basic needs like food/housing |  |  |  |  | .064 |
| Yes | 259 (19%) | 135 (17.3%) | 172 (22.1%) | 63 (17%) |  |
| No | 1,104 (81%) | 645 (82.7%) | 606 (77.9%) | 308 (83%) |  |
| Parent or guardian divorced or separated |  |  |  |  | .173 |
| Yes | 378 (27.7%) | 236 (30.3%) | 231 (29.7%) | 91 (24.5%) |  |
| No | 985 (72.3%) | 544 (69.7%) | 547 (70.3%) | 280 (75.5%) |  |
| Parent or guardian died |  |  |  |  | .272 |
| Yes | 99 (7.3%) | 45 (5.8%) | 41 (5.3%) | 23 (6.2%) |  |
| No | 1,264 (92.7%) | 735 (94.2%) | 737 (94.7%) | 348 (93.8%) |  |
| Parent or guardian served time in jail or prison |  |  |  |  | .003 |
| Yes | 140 (10.3%) | 87 (11.2%) | 71 (9.1%) | 17 (4.6%) |  |
| No | 1,223 (89.7%) | 693 (88.8%) | 707 (90.9%) | 354 (95.4%) |  |
| Witnessed domestic violence |  |  |  |  | .360 |
| Yes | 93 (6.8%) | 57 (7.3%) | 41 (5.3%) | 22 (5.9%) |  |
| No | 1,270 (93.2%) | 723 (92.7%) | 737 (94.7%) | 349 (94.1%) |  |
| Victim or witness of neighborhood violence |  |  |  |  | .045 |
| Yes | 61 (4.5%) | 52 (6.7%) | 47 (6%) | 13 (3.5%) |  |
| No | 1,302 (95.5%) | 728 (93.3%) | 731 (94%) | 358 (96.5%) |  |
| Lived with anyone with mental health problems |  |  |  |  | .821 |
| Yes | 98 (7.2%) | 61 (7.8%) | 52 (6.7%) | 29 (7.8%) |  |
| No | 1,265 (92.8%) | 719 (92.2%) | 726 (93.3%) | 342 (92.2%) |  |
| Lived with anyone with alcohol/drug problems |  |  |  |  | .470 |
| Yes | 103 (7.6%) | 62 (7.9%) | 52 (6.7%) | 21 (5.7%) |  |
| No | 1,260 (92.4%) | 718 (92.1%) | 726 (93.3%) | 350 (94.3%) |  |
| Cumulative ACEs | 0.90 (1.28) | 0.94 (1.28) | 0.91 (1.19) | 0.75 (1.14) | .105 |
| *Note:* *p* = *p*-value for Chi-Square Test of Independence. Higher quartiles associated with greater state-level opportunity. | | | | | |

| **Supplemental Table S7**  *Associations Between Individual ACEs Exposures and State-level Opportunity Quantiles: Hispanic (n = 8,370).* | | | | | |
| --- | --- | --- | --- | --- | --- |
| ACEs indicators | Bottom 25% | 25% to 50% | 50% to 75% | Top 25% | *p* |
|  | (*n* = 2,232) | (*n* = 1,496) | (*n* = 3,100) | (*n* = 1,542) |  |
| Difficulties with basic needs like food/housing |  |  |  |  | .671 |
| Yes | 353 (15.8%) | 242 (16.2%) | 508 (16.4%) | 267 (17.3%) |  |
| No | 1,879 (84.2%) | 1,254 (83.8%) | 2,592 (83.6%) | 1,275 (82.7%) | |
| Parent or guardian divorced or separated |  |  |  |  | .721 |
| Yes | 564 (25.3%) | 359 (24%) | 757 (24.4%) | 392 (25.4%) |  |
| No | 1,668 (74.7%) | 1,137 (76%) | 2,343 (75.6%) | 1,150 (74.6%) | |
| Parent or guardian died |  |  |  |  | .061 |
| Yes | 66 (3%) | 58 (3.9%) | 92 (3%) | 34 (2.2%) |  |
| No | 2,166 (97%) | 1,438 (96.1%) | 3,008 (97%) | 1,508 (97.8%) | |
| Parent or guardian served time in jail or prison |  |  |  |  | .041 |
| Yes | 141 (6.3%) | 99 (6.6%) | 158 (5.1%) | 107 (6.9%) |  |
| No | 2,091 (93.7%) | 1,397 (93.4%) | 2,942 (94.9%) | 1,435 (93.1%) | |
| Witnessed domestic violence |  |  |  |  | .084 |
| Yes | 129 (5.8%) | 84 (5.6%) | 141 (4.5%) | 94 (6.1%) |  |
| No | 2,103 (94.2%) | 1,412 (94.4%) | 2,959 (95.5%) | 1,448 (93.9%) | |
| Victim or witness of neighborhood violence |  |  |  |  | .431 |
| Yes | 81 (3.6%) | 68 (4.5%) | 137 (4.4%) | 68 (4.4%) |  |
| No | 2,151 (96.4%) | 1,428 (95.5%) | 2,963 (95.6%) | 1,474 (95.6%) | |
| Lived with anyone with mental health problems |  |  |  |  | <.001 |
| Yes | 202 (9.1%) | 139 (9.3%) | 210 (6.8%) | 160 (10.4%) |  |
| No | 2,030 (90.9%) | 1,357 (90.7%) | 2,890 (93.2%) | 1,382 (89.6%) | |
| Lived with anyone with alcohol/drug problems |  |  |  |  | .059 |
| Yes | 206 (9.2%) | 139 (9.3%) | 239 (7.7%) | 151 (9.8%) |  |
| No | 2,026 (90.8%) | 1,357 (90.7%) | 2,861 (92.3%) | 1,391 (90.2%) | |
| Cumulative ACEs | 0.78 (1.29) | 0.79 (1.29) | 0.72 (1.14) | 0.83 (1.29) | .035 |
| *Note:* *p* = *p*-value for Chi-Square Test of Independence. Higher quartiles associated with greater state-level opportunity. | | | | | |

| **Supplemental Table S8**  *Spearman Bivariate Correlations Between Cumulative and Individual ACEs Indictors.* | | | | |
| --- | --- | --- | --- | --- |
| Variables | Cumulative ACEs | | | |
|  | Overall  *(N = 46,395)* | non-Hispanic White  *(n = 34,733)* | non-Hispanic Black  *(n = 3,292)* | Hispanic  *(n = 8,370)* |
| Difficulties with basic needs like food/housing | .516 (<.001) | .501 (<.001) | .520 (<.001) | .547 (<.001) |
| Parent or guardian divorced or separated | .730 (<.001) | .736 (<.001) | .675 (<.001) | .726 (<.001) |
| Parent or guardian died | .261 (<.001) | .258 (<.001) | .301 (<.001) | .242 (<.001) |
| Parent or guardian served time in jail or prison | .418 (<.001) | .412 (<.001) | .456 (<.001) | .417 (<.001) |
| Witnessed domestic violence | .398 (<.001) | .394 (<.001) | .409 (<.001) | .411 (<.001) |
| Victim or witness of neighborhood violence | .318 (<.001) | .307 (<.001) | .348 (<.001) | .340 (<.001) |
| Lived with anyone with mental health problems | .507 (<.001) | .539 (<.001) | .403 (<.001) | .454 (<.001) |
| Lived with anyone with alcohol/drug problems | .531 (<.001) | .557 (<.001) | .421 (<.001) | .494 (<.001) |
